# Improving the Diet for the Rearing of *Glossina brevipalpis* Newstead and *Glossina austeni* Newstead: Blood Source and Collection – Processing – Feeding Procedures

**DOI:** 10.1371/journal.pone.0168799

**Published:** 2016-12-22

**Authors:** Chantel J. De Beer, Gert J. Venter, Marc J. B. Vreysen

**Affiliations:** 1 Agricultural Research Council-Onderstepoort Veterinary Institute (ARC-OVI), Parasites, Vectors & Vector-borne Diseases, South Africa; 2 Department of Zoology and Entomology, Faculty of Natural and Agricultural Sciences, University of the Free State, South Africa; 3 Department of Veterinary Tropical Diseases, Faculty of Veterinary Science, University of Pretoria, South Africa; 4 Joint FAO/IAEA Division of Nuclear Techniques in Food and Agriculture, Insect Pest Control Laboratory, Austria; New Mexico State University, UNITED STATES

## Abstract

One of the challenges to maintain tsetse fly (Diptera: Glossinidae) colonies is the sustainable supply of high quality blood meals. The effect of using anticoagulants during collection of the blood, the addition of phagostimulants to the blood meals as well as using mixtures of bovine and porcine blood in different proportions for feeding on colony productivity was assessed. Defibrinated bovine blood was found to be suitable to maintain both the *Glossina brevipalpis* Newstead and *Glossina austeni* Newstead colonies. Blood collected with the anticoagulants sodium citrate, citric sodium combination, citrate phosphate dextrose adenine and citric acid did not affect colony performance of both species. Defibrinated bovine and porcine blood in a 1:1 ratio or the feeding of either bovine or porcine blood on alternating days improved pupae production of *G*. *austeni* and can be used to enhance colony growth. Bovine blood is appropriate to maintain *G*. *brevipalpis* colonies, however, feeding either bovine or porcine blood on alternating days did improve productivity. Adding the phagostimulants inosine tri-phosphate, cytosine mono-phosphate and guanosine mono-phosphate to the blood at a concentration of 10^−4^ M improved pupae production of the *G*. *brevipalpis* colony. The addition of adenosine tri-phosphate and inosine tri-phosphate improved the performance of the *G*. *austeni* colony. Decisions on the most suitable rearing diet and feeding protocols will not only depend on the biological requirements of the species but also on the continuous supply of a suitable blood source that can be collected and processed in a cost-effective way.

## Introduction

Tsetse flies (Diptera: Glossinidae) are responsible for the cyclical transmission of trypanosome parasites that affect livestock (nagana) and humans (sleeping sickness) throughout sub-Saharan Africa. After an epidemiological silence of nearly 33 years, an outbreak of nagana in the north eastern parts of the KwaZulu-Natal Province in 1990, showed the devastating socio-economic impact of tsetse-transmitted nagana on these largely rural communities of South Africa [1]. In South Africa nagana is restricted to the north eastern parts of the KwaZulu-Natal Province, where an estimated 250000 cattle are at risk of contracting the disease [2]. Ever since the successful and sustainable eradication of a *Glossina pallidipes* Austen population from Zululand in the 1950’s, the two remaining tsetse species *Glossina brevipalpis* Newstead and *Glossina austeni* Newstead have always been considered of minor epidemiological importance [3–7]. The outbreak of nagana in 1990 showed that this assumption was a fallacy [8].

An area-wide integrated pest management (AW-IPM) program with a sterile insect technique (SIT) component was proposed as a strategy to establish a tsetse free South Africa [2]. This strategy consisted of a phase of tsetse population reduction using the sequential aerosol technique (SAT) followed by the release of sterile males to eradicate relic pockets. The SIT requires the production of the target insect in large numbers, the sterilisation of the males with ionising radiation [9] followed by the sequential and area-wide release of these males in numbers sufficient to outcompete their wild counterparts for mating with fertile females [10,11]. A mating of a sterile male with a virgin wild female will result in no progeny, which will lead to population reduction and the eventual eradication of the target population [11].

A principal component of the SIT is the sustainable production and maintenance of large numbers of high quality insects [12]. Both male and female tsetse flies are obligate blood feeders and one of the main challenges in tsetse fly colony maintenance is the sustained supply of high quality blood. Tsetse flies reproduce by adenotrophic viviparity, making the adult and larval stages dependent on the same source of food and insufficient nutrition of the female fly will lead to abortions [13,14]. Increased abortion rates and reduced productivity resulting from an inadequate diet will hamper colony growth, which is already inherently low due to the slow reproductive cycle of tsetse flies.

The maintenance of live animals for the mass-rearing of tsetse flies will be costly, labour intensive and can even be considered as unethical. The development of *in vitro* feeding techniques was a breakthrough making the mass-rearing of tsetse flies more cost effective [15]. The logistics of obtaining sterile, high quality blood remain challenging. An added complication is the variation in nutritional value of the collected blood which is influenced by genetic, environmental, chemical and physical factors of the host animal [16]. Chemicals and microbiological contamination during collection, handling and storage will also play a role [16]. Viable productive colonies can only be obtained when standard blood collection procedures are developed, implemented and adhered to, as this will ensure the continuous supply of a quality product comparable to fresh blood collected from donor animals.

In preparation for the proposed AW-IPM program in South Africa, laboratory colonies of *G*. *brevipalpis* and *G*. *austeni* were established at the Agricultural Research Council-Onderstepoort Veterinary Institute (ARC-OVI) in Pretoria, South Africa in 2002. These colonies of *G*. *brevipalpis* and *G*. *austeni* were established using seed material from the Tsetse and Trypanosomiasis Research Institute (TTRI) (now named Vector & Vector-Borne Diseases Research Institute), Tanga, Tanzania and the Entomology Unit of the FAO/IAEA’s Laboratories in Seibersdorf, Austria (now called the FAO/IAEA Insect Pest Control Laboratory), respectively. *Glossina brevipalpis* was originally colonised in the mid-1980s from flies collected in the Kibwezi Forest in Kenya [17]. The original *G*. *austeni* colony was established at the TTRI in September 1982 from pupae collected in the Jozani Forest, Unguja Island of Zanzibar. Both these colonies were initially maintained on rabbits before an *in vitro* feeding system was introduced [17,18].

In view of the importance of providing high quality blood to tsetse colonies, optimisation of blood collection procedures and the development and validation of methods that may improve colony performance should be part of any colony maintenance program. The process of collecting blood at the abattoir could be simplified by using anticoagulants as a surrogate for mechanical stirring. We therefore tested the effect of mixing various anticoagulants on the nutritional value of the blood.

It is known that the host preference of *G*. *brevipalpis* and *G*. *austeni* in nature may include, in addition to bovines, smaller mammals such as bush pigs [19]. Porcine- and bovine blood, and various combinations thereof, were therefore evaluated as rearing diets for these two species. Finally, it is also known that phagostimulants may enhance blood intake of female tsetse flies and hence, increase productivity. We therefore tested the effects of phagostimulants on the performance of the colonies.

## Materials and Methods

### Ethical statement

Materials used in the study posed no health risk to researchers and no vertebrate animals were harmed. Permission to do research in terms of Section 20 of the animal diseases act (Act no. 35 of 1984) of South Africa has been granted for tsetse fly colony maintenance, Ref 12/11/1/1.

### Tsetse colonies

Since the initiation of the colonies of *G*. *brevipalpis* and *G*. *austeni* at the ARC-OVI in 2002, they have been maintained on defibrinated bovine blood, using an artificial *in vitro* membrane feeding system [20–22]. These colonies have been maintained under standard rearing conditions of 23–24°C, 75–80% RH and subdued/indirect lighting with a 12h light / 12h dark photoperiod [21,22]. At the end of 2015, the *G*. *brevipalpis* colony consisted of 13000 reproducing females that produced 7000 pupae weekly and the *G*. *austeni* colony had a size of 16000 reproducing females that produced 5000 pupae weekly. The total colony size including both males and females of both species was 37000 flies at the end of 2015.

### Blood collection and processing

#### Porcine and bovine blood collected from commercial abattoirs for routine colony maintenance

The two colonies at the ARC-OVI are currently maintained on cattle blood collected at a commercial abattoir, situated outside the tsetse fly and nagana infected area, where cattle are slaughtered for human consumption. Although the veterinary history of these animals is unknown, all animals slaughtered are accompanied by a health certificate stating they are healthy and of good quality. This abattoir does not slaughter for disease control purposes.

Collection of blood was carried out as follows: cattle were stunned, then suspended by their hind legs and their throat slit. The swathe of blood was directed into a bucket and transferred to 40 L containers where it was defibrinated for 10 to 15 minutes using a custom-made stainless steel electric paddle stirrer. The clotted fibrin was removed by hand and the blood pooled in a 500 L container after which it was divided into 5 L canisters and stored at -20°C [23].

Porcine blood was obtained from pigs with a known veterinary history, kept at the Agricultural Research Council-Animal Improvement Institute for breeding and experimental purposes. All housing and experimental work on pigs have been approved by the animal ethics committee of the Agricultural Research Counsel. The pigs were slaughtered at the BonAccord Abattoir south of Pretoria. Animals were stunned, the jugular vein exposed and severed, and the blood collected in a 3 L sterilized glass jar containing glass beads, and defibrinated by agitating for 10 to 15 minutes before it was dispensed into 0.3 L containers and stored at -20°C.

The bovine and porcine blood collected from the abattoirs was irradiated when frozen with 2 kGy at Synergy Heath, a commercial irradiation facility and stored in 5 L (bovine) or 0.3 L (porcine) containers at -20°C. All collected blood was tested for bacterial contamination before being used for tsetse fly feeding. Contaminated blood samples were appropriately discarded [22].

#### Bovine blood collected with a closed sterile system

As the veterinary history of the cattle slaughtered at the commercial abattoir was not known, cattle from a closed quarantined herd with a known veterinary background were used for evaluation of the anticoagulants and phagostimulants. For this purpose, blood was taken from the jugular vein using a trocar and cannula. Blood was drained directly (in a closed sterile system) into a sterilized 3 L glass jar containing one of the following anticoagulants; acid citrate dextrose (ACD), sodium citrate, a combination of citric acid and sodium citrate, citrate phosphate dextrose adenine (CPDA) or citric acid. For the evaluation of the phagostimulants defibrinated blood was used. Blood was collected in 3 L glass jars containing only glass beads and agitated for 10 to 15 minutes. The blood collected with anticoagulants and defibrination were then decanted into 20 mL containers and stored at -20°C.

### Assessment of suitability of blood source as a maintenance diet

Bovine and porcine blood and mixtures thereof were evaluated as diets for the routine maintenance of *G*. *brevipalpis* and *G*. *austeni* colonies. In a first set of experiments, productivity of both species was assessed when fed on a single blood source (bovine or porcine) or in one of the following combinations (75% bovine / 25% porcine, 50% bovine / 50% porcine, and 25% bovine / 75% porcine).

In a second set of experiments productivity of both species was assessed when flies were fed on a single blood source that was alternated between days according to the following schedule:

- One day bovine followed by four days of porcine blood,
- Three days bovine followed by three days of porcine blood,
- Four days bovine followed by one-day of porcine blood.

Bovine blood was collected using both the abattoir and closed sterile system and porcine blood using the abattoir system.

### Phagostimulation

The nucleotides adenosine tri-phosphate (ATP), inosine tri-phosphate (ITP), cytosine mono-phosphate (CMP) and guanosine mono-phosphate (GMP) were the phagostimulants evaluated. An amount of 0.055 g of each compound was diluted in 10 mL distilled water. The dilutions were kept at -20°C and used within three days. Only 0.02 mL of each solution (concentration of 10^−4^ M) was used for 20 mL of bovine blood collected with the closed sterile system.

### Bioassay

Female fly survival (percentage alive on day 18 and 30 respectively), fecundity (number of pupae per mature female at day 18) and pupal size are three essential comprehensive parameters routinely used for assessing colony performance [20]. These parameters are combined in a formula that calculates a Quality Factor (QF) of the blood as a comprehensive indicator for colony production [21,22]. A QF above 1 is considered acceptable and indicates that the blood diet is suitable for colony maintenance [21].

Blood quality was evaluated using the standardised bioassay [22] whereby productivity of flies fed daily on the selected diet for 30 days using an artificial membrane feeding system was assessed and that enabled the QF of the selected diet to be calculated [22,24].

The protocol of the bioassay consisted of mating 30 three-day-old females with 30 eight-day-old males at a 1:1 ratio. Males and females were kept together for four days under standard colony conditions (23–24°C, 75–80% RH and subdued/indirect lighting). After removing the male flies, female survival, pupae production and abortions (i.e. expelled eggs and immature larval stages) were monitored daily for 30 days [21,22].

All pupae produced were mechanically sorted into one of five class sizes (A-E). The sorter was calibrated according to the standards established by the FAO/IAEA. For *G*. *austeni* the measurements ranged between 2.3 mm (A) and 3.0 mm (E), and for *G*. *brevipalpis* between 3.5 mm (A) and 4.3 mm (E). The corresponding weight (mg) of the different pupal size classes were A (<16), B (16 –<19), C (19 –<21), D (21 –<23), E (≥23) for *G*. *austeni* and A (<56), B (56 –<68), C (68 –<76), D (76 –<84), E (≥84) for *G*. *brevipalpis*.

After 30 days, all surviving females were dissected to determine their reproductive status (presence/absence of egg/larvae in the uterus and spermathecae fill). All bioassays were replicated four times. The QFs were calculated using the formula [21,22]:

$$Qf=\frac{FS30+PT+0.3(PB)+0.4(PC)+0.5(PD)+0.6(PE)+0.3(E+I)+0.6(II+III)-0.2(PA)-0.5(AB)-BO}{FS30+FS18}$$

Where:

**Table:** 

QF	Quality Factor
FS18	Female flies surviving on day 18
FS30	Female flies surviving on day 30
PA	No. of pupae in size class A
PB	No. of pupae in size class B
PC	No. of pupae in size class C
PD	No. of pupae in size class D
PE	No. of pupae in size class E
PT	Total pupae
AB	Abortions
E&I	Eggs or first instar larvae *in utero*
II&III	Second or third instar larvae *in utero*
BO	Blockage of the oviducts or other reproductive abnormality

### Statistical analysis

Data were analysed using the statistical programs GraphPad Instat [25] and GenStat [26]. The experiments were designed as a randomised block design in four blocks. Differences in survival rate and QF values between treatments were determined with an analysis of variance (ANOVA). The data were normally distributed with homogeneous treatment variances. Treatment means were separated using Fishers' protected *t*-test least significant difference (LSD) at the 5% level of significance [27], if the F-probability from the ANOVA was significant at 5%.

## Results

### Anticoagulants

A total of 120 females of each species were offered blood that had been mixed during collection with one of the following anticoagulants: acid citrate dextrose (ACD), sodium citrate, a combination of citric acid and sodium citrate, citrate phosphate dextrose adenine (CPDA) or citric acid (four replicates) (Table 1).

**Table 1:** Anticoagulants tested for their potential use in blood collection, as opposed to defribination, for *Glossina brevipalpis* and *Glossina austeni* rearing diets. Numbers followed by an * indicate significant differences between the anticoagulant and the defribinated blood for each species. Quality Factor values (QF) denoted by a different alphabetical letter indicate a significantly differences for *G*. *austeni*. Testing was done at the 5% level.

	No. of mature females		Pupae produced	Fecundity	Pupal size classes					QF	Uterus					Insemination	Spermathecae fill			
											Recently ovulated egg	Empty due to abortion	Viable instar larvae							
	Day 18	Day 30			A	B	C	D	E				I	II	III		0.25	0.5	0.75	1
*G*. *brevipalpis*																				
Defibrination	117	117	80	0.053	4	6	14	21	35	1.11	13	31	29	20	24	1.00	7	49	49	12
Acid citrate dextrose	118	111	71	0.047	11	24	21	9	6	0.93	23	36	33	11	8	1.00	5	42	56	8
Sodium citrate	116	114	82	0.055	15	16	18	23	10	1.07	30	26	25	22	10	0.95	15	42	46	4
Citric acid & sodium citrate	109	106*	83	0.059	1	5	17	32	28	1.12	6	33	48	12	5	1.00	0	60	39	6
Citrate phosphate dextrose adenine	108	101*	68	0.050	3	7	15	21	22	1.05	21	24	31	16	9	0.98	12	52	35	2
Citric acid	98	96*	77	0.061	4	4	14	24	31	1.17	6	24	42	14	9	1.00	0	48	43	4
*G*. *austeni*																				
Defibrination	115	114	100	0.067	12	14	28	31	15	1.31^**a**^	27	7	8	32	39	0.97	16	58	29	5
Acid citrate dextrose	111	103*	82	0.059	13	18	22	22	7	1.16^**bc**^	28	11	14	19	31	1.00	27	37	35	4
Sodium citrate	98	81*	57	0.049	9	10	22	11	5	1.03^**c**^	23	12	3	17	25	0.99	25	30	16	8
Citric acid & Sodium citrate	115	102*	88	0.062	6	6	24	34	18	1.22^**ab**^	25	9	10	28	23	0.98	9	60	29	2
Citrate phosphate dextrose adenine	108	96*	86	0.065	4	6	25	34	17	1.26^**ab**^	15	9	19	28	24	0.97	7	61	18	7
Citric acid	106	102*	86	0.063	8	8	24	35	10	1.22^**ab**^	15	16	23	25	22	0.99	18	50	26	5

Survival on day 30 of both *G*. *brevipalpis* (98%) and *G*. *austeni* (95%) was the highest for female flies that had fed on defibrinated blood (Table 1). The survival of *G*. *brevipalpis* females that had fed on blood collected with ACD (93%) (P = 0.14) or sodium citrate (95%) (P = 0.50) was not significantly different from that of female flies fed on defibrinated blood. However, survival of *G*. *brevipalpis* females fed on blood collected with the citric sodium combination (88%), CPDA (84%) or citric acid (80%) was significantly lower (P *≤* 0.01 in all cases) than that of female flies fed on defibrinated blood (Table 1).

Survival of *G*. *austeni* females that fed on blood collected with ACD (86%), citric sodium combination (85%), citric acid (85%), sodium citrate (68%) or CPDA (80%) was significantly lower (P ≤ 0.02 in all cases) than that of female flies fed on defibrinated blood (95%) (Table 1).

*Glossina brevipalpis* females fed on blood collected with the citric sodium combination (0.059 pupae per mature female day) or citric acid (0.061 pupae per mature female day) showed the highest fecundity (Table 1) and these females also produced the highest proportion of large pupae (pupal class C > 68 mg), i.e. 87% and 93%, respectively. Feeding *G*. *brevipalpis* females with blood collected with ACD reduced the fecundity to 0.053 pupae per mature female day and these females also produced the smallest pupae (Table 1).

The fecundity of *G*. *austeni* females fed on defibrinated blood as well as blood collected with CPDA or citric acid was above 0.062 pupae per mature female day. Those that had fed on blood mixed with CPDA produced the largest pupae (88% > 19 mg). The lowest fecundity of 0.049 pupae per mature female day was observed for *G*. *austeni* females fed on blood collected with sodium citrate. Similar to *G*. *brevipalpis*, the smallest pupae were produced by females that fed on blood collected with ACD (Table 1).

Insemination rate of all surviving females on day 30 was higher than 95% and the spermathecae fill was above 0.5 for most of the female flies for both species, irrespective of the treatment (Table 1).

Despite significant differences in productivity of female flies fed on blood mixed with different anticoagulants, the QF values obtained were above 1 for both species for all anticoagulants tested (as well as for the defibrinated blood) (Table 1). The survival rate of *G*. *brevipalpis* females that were fed with the various anticoagulants showed significant variations, but the QF values of the blood did not differ from each other or from that of that of defibrinated blood (Table 1). For *G*. *austeni* only the low QF values obtained with sodium citrate (1.03) or ACD (1.16) were significantly different form that of flies that had fed on defibrinated blood (1.31) (Table 1).

### Blood source and feeding protocols

A total of 120 females of each species were each fed on blood using two feeding regimes during four replicates. The regimes consisted of offering pure bovine or porcine blood or bovine and porcine blood mixed in different proportions or using different blood source sequences during a six-day cycle.

The highest survival rate on day 30 of both *G*. *brevipalpis* (83%) and *G*. *austeni* (72%) females was obtained with the 50% / 50% bovine/porcine combination (Table 2). Feeding only bovine blood reduced the overall survival rate to 53% and 61% for *G*. *brevipalpis* and *G*. *austeni*, respectively. The lowest survival rates, 35% for *G*. *brevipalpis* and 32% for *G*. *austeni*, were obtained with the 25% / 75% bovine / porcine combination. *Glossina brevipalpis* females that fed on pure bovine blood survived significantly longer as compared with female flies that had fed on pure porcine blood (P ≤ 0.01) as well as all other diet combinations (P < 0.01) (Table 2). *Glossina austeni* females fed on pure porcine and the 25% bovine / 75% porcine combination survived significantly longer (P < 0.01) than female flies that had fed on pure bovine blood (Table 2).

**Table 2:** Bovine/porcine blood combinations tested for their potential use as rearing diet for *Glossina brevipalpis* and *Glossina austeni*. Numbers followed by an * indicate a significant difference between the bovine blood (control) and the various combinations for each species and each group at the 5% level. Quality Factor values (QF) denoted by a different alphabetical letter indicate significant differences for each species. Testing was done at the 5% level. (# = days).

	No. of mature females		Pupae produced	Fecundity	Pupal size classes					QF	Uterus					Insemination	Spermathecae fill			
											Recently ovulated egg	Empty due to abortion	Viable instar larvae							
	Day 18	Day 30			A	B	C	D	E				I	II	III		0.25	0.5	0.75	1
**Premix combination diet**																				
*G*. *brevipalpis*																				
Bovine (bov)	80	63*	20	0.020	2	2	5	5	6	1.11^**a**^	22	18	9	5	9	0.98	10	31	20	1
Porcine (por)	112	96*	41	0.031	7	5	13	8	8	0.73^**c**^	15	24	30	16	3	1.00	0	36	54	6
25%bov/75%por	60	38*	22	0.035	4	2	7	6	3	0.54^**c**^	10	17	3	3	5	0.88	3	13	20	1
75%bov/25%por	100	85*	42	0.032	1	4	9	13	11	0.79^**bc**^	27	29	16	7	3	0.95	19	20	39	3
50%bov/50%por	113	99*	55	0.039	8	8	14	19	6	0.95^**ab**^	11	14	17	36	8	0.96	18	24	43	1
*G*. *austeni*																				
Bovine (bov)	83	73	36	0.035	6	15	9	5	1	0.84^**a**^	27	20	9	11	6	0.97	22	33	23	3
Porcine (por)	105	93*	56	0.040	29	9	15	2	1	0.86^**a**^	44	17	15	10	4	1.00	20	31	37	2
25%bov/75%por	60	38*	13	0.022	5	2	3	2	1	0.56^**b**^	10	17	2	4	5	0.88	3	13	20	1
75%bov/25%por	96	85	58	0.050	13	10	17	13	5	0.93^**a**^	27	28	5	18	3	0.95	19	20	39	3
50%bov/50%por	105	86	63	0.052	17	10	15	14	7	0.99^**a**^	14	12	31	20	7	0.96	18	24	43	1
**Alternating diet**																				
*G*. *brevipalpis*																				
Bovine (bov)	118	117	91	0.060	5	6	16	34	30	1.13	30	35	24	23	5	1.00	0	57	52	8
Porcine (por)	118	118	88	0.057	6	14	19	27	22	1.11	43	24	28	7	7	0.99	5	62	43	6
bov(1#)-por(4#)	118	112	95	0.063	5	8	26	24	32	1.19	50	9	16	7	7	0.95	11	48	23	7
bov(4#)-por(1#)	117	116*	92	0.061	5	5	24	26	32	1.17	37	16	28	12	6	0.96	26	43	28	3
bov(3#)-Por(3#)	112	105*	95	0.067	6	9	21	26	33	1.21	35	15	20	16	3	0.95	11	37	29	4
*G*. *austeni*																				
Bovine (bov)	104	99	79	0.059	8	13	18	16	24	1.17	48	11	19	16	5	0.97	14	49	25	8
Porcine (por)	88	87	66	0.058	9	12	24	11	10	1.08	36	22	15	9	5	1.00	11	49	22	5
bov(1#)-por(4#)	93	91	72	0.060	2	6	12	26	26	1.21	50	8	16	8	7	0.95	11	48	23	7
bov(4#)-por(1#)	104	103	82	0.061	2	8	18	22	32	1.19	37	16	28	9	6	0.96	26	43	28	3
bov (3#)-por(3#)	95	94	68	0.055	4	7	12	21	24	1.13	35	15	19	16	3	0.95	11	37	29	4

Overall fecundity was very low (Table 2) with the highest values recorded for *G*. *brevipalpis* (0.039 pupae per mature female day) as well as *G*. *austeni* (0.052 pupae per mature female day) that had fed on the 50% / 50% combination diet. Female *G*. *brevipalpis* (87%) and *G*. *austeni* (60%) that fed on the 75% bovine / 25% porcine combination produced the highest percentage of pupae of size class C and above (Table 2). Insemination rate was for both species and for all treatments ≥ 0.88 and the spermathecae fill was in most cases between 0.5 and 0.75 (Table 2). All QF values for the premix combinations used to feed *G*. *brevipalpis* were below 1, except for those that fed on pure bovine blood (Table 2). Except for flies that fed on the 50% bovine / 50% porcine combination, the QF values for *G*. *brevipalpis* females of all other treatments were significantly lower (P < 0.01) as compared with female flies that had fed on pure bovine blood (Table 2).

The QF value of *G*. *austeni* females that had fed on pure bovine blood was significantly different (P < 0.01) from the females that fed on the 25% bovine / 75% porcine combination.

In this specific evaluation the QF values obtained for *G*. *brevipalpis* and *G*. *austeni* with the bovine blood control were lower than normal which may indicate that the overall quality of the bovine as well as porcine blood used in this trial was low.

During the second feeding regime evaluation, blood from a single host was offered to the flies in different sequences during a 6-day cycle. Survival of the *G*. *brevipalpis* females on day 30 ranged from 98% for females fed on the pure bovine or porcine blood, to 88% for flies fed on the bovine (3 days)–porcine (3 days) sequence (Table 2). Survival of *G*. *brevipalpis* females fed on the bovine (3 days)–porcine (3 days) and bovine (4 days)–porcine (1 day) sequence (P < 0.01 in both cases) was significantly lower as compared with those fed on pure bovine blood. Survival of *G*. *austeni* females ranged between 83% for flies that had fed on pure bovine blood to 73% for flies fed on the pure porcine blood. Survival of the *G*. *austeni* females fed on pure bovine blood was similar to that of females fed on any of the other combinations (Table 2).

The insemination rates for both species were above 0.95 and most females showed a spermathecae fill in the 0.5 or 0.75 class (Table 2). *Glossina brevipalpis* females that had fed on the bovine (3 days)–porcine (3 days) combination had a fecundity of 0.067 pupae per mature female day, whereas fecundity was reduced to 0.057 pupae per mature female day for female flies that had fed on the pure porcine blood (Table 2). *Glossina brevipalpis* females that fed on pure porcine blood produced the smallest pupae (Table 2), whereas the largest pupae were produced by female flies fed on the bovine (4 days)–porcine (1 day) combination. The *G*. *austeni* females that had fed on the bovine (4 days)–porcine (1 day) combination produced the highest number of pupae (fecundity of 0.061 pupae per mature female day) and those fed on the bovine (3 days)–porcine (3 days) produced the least (0.055 pupae per mature female day). The *G*. *austeni* females that were offered blood in the combination diets produced larger pupae than those fed on a single source diet (Table 2). All the different feeding regimes resulted in QF values above 1 for both species with no significant differences between treatments (Table 2).

### Phagostimulants

Bovine blood was spiked with phagostimulants to assess potential stimulated feeding responses that could increase overall colony productivity. A total of 120 females of each species were fed on blood mixed with each stimulant during four replicates.

The survival rate of *G*. *brevipalpis* (94%) females that fed on bovine blood containing ITP was equal to that of females that fed on pure bovine (control) blood (Table 3). Blood with ITP or GMP improved the survival rate (94% and 92% respectively) of *G*. *austeni* as compared with flies fed on the pure bovine diet only (72%) (Table 3). The lowest survival rates, obtained for *G*. *brevipalpis* (76%) and *G*. *austeni* (49%) fed on blood with ATP, were significantly different (P < 0.01) from those of flies fed on the pure bovine blood (Table 3).

**Table 3:** Blood mixed with different phagostimulants (nucleotides adenosine tri-phosphate (ATP), inosine tri-phosphate (ITP), cytosine mono-phosphate (CMP) and guanosine mono-phosphate (GMP)) tested for their potential use as rearing diet for *Glossina brevipalpis* and *Glossina austeni*. Quality Factor values (QF) denoted by a different alphabetical letter indicate a significantly differences for *G*. *austeni* at the 5% level.

	No. of mature females		Pupae produced	Fecundity	Pupal size classes					QF	Uterus					Insemination	Spermathecae fill			
											Recently ovulated egg	Empty due to abortion	Viable instar larvae							
	Day 18	Day 30			A	B	C	D	E				I	II	III		0.25	0.5	0.75	1
*G*. *brevipalpis*																				
Control	115	113	83	0.056	1	1	20	23	38	1.09	38	35	22	7	11	0.87	39	17	8	3
ATP	92	91	57	0.048	2	6	16	21	12	1.02	26	21	9	13	9	0.87	22	14	13	0
ITP	116	113	84	0.056	3	3	19	21	38	1.11	47	28	20	10	6	0.89	42	25	6	4
CMP	109	107	75	0.054	0	6	20	24	25	1.10	48	21	26	6	5	0.93	41	17	6	3
GMP	116	110	65	0.044	1	5	10	22	27	1.10	44	21	21	18	3	0.94	50	17	14	2
*G*. *austeni*																				
Control	96	86	66	0.057	9	17	20	17	3	1.06^**ab**^	39	10	20	4	7	0.99	11	38	30	6
ATP	71	59	56	0.067	3	7	20	23	3	1.18^**a**^	22	7	14	13	0	0.98	9	22	22	4
ITP	103	94	74	0.058	7	8	29	21	9	1.08^**ab**^	42	10	25	6	4	0.99	5	57	21	4
CMP	89	75	52	0.049	16	9	19	7	1	0.98^**b**^	41	5	17	6	6	0.99	8	40	21	4
GMP	103	96	72	0.056	6	4	27	20	15	1.14^**a**^	50	5	18	13	4	1.00	6	60	21	7

The insemination rate for *G*. *brevipalpis* ranged from 0.87 for flies fed on pure bovine blood to 0.94 for flies fed on blood containing GMP. The spermathecae fill of *G*. *brevipalpis* females was low for all the diets and was mostly in the 0.25 or 0.5 class range (Table 3). More than 98% of *G*. *austeni* females were inseminated irrespective of the treatment and most females had a spermathecae fill between 0.5 and 0.75 (Table 3). *Glossina brevipalpis* females that fed on pure bovine blood or blood mixed with ITP produced the highest number of pupae with a fecundity of 0.056 pupae per mature female day (Table 3). *Glossina brevipalpis* females fed on pure bovine blood produced the largest (98% in the C class and above) pupae (Table 3), whereas flies that were fed blood containing ATP produced the smallest pupae (86% in the C class and above). *Glossina austeni* females fed on blood containing ATP, ITP and GMP produced the most as well as the largest pupae (Table 3).

All of the quality control tests with phagostimulant-treated blood as well as the pure bovine blood diets resulted in QF values above 1 for *G*. *brevipalpis* (Table 3) with no significant differences among the treatments (P = 0.64). For *G*. *austeni*, the QF value of the diet spiked with CMP (0.98) was below 1 (Table 3) and this was significantly different (P = 0.04) from the values obtained for GMP (1.14) and ATP (1.18), which was the highest QF value obtained (Table 3).

## Discussion

High quality blood as a rearing diet is critical to ensure the growth and sustainability of tsetse fly colonies [21]. Obtaining sterile, high quality blood required for a tsetse fly *in vitro* feeding system can be challenging, although, sterile, freshly frozen, defibrinated or heparinized blood used with a silicon membrane is considered the most efficient way of maintaining larger tsetse colonies [28,29]. In the present study, this diet was also found to be the most suitable for maintaining the *G*. *brevipalpis* and *G*. *austeni* colonies at the ARC-OVI. Additionally, our study showed that bovine blood collected with the anticoagulants sodium citrate, citric sodium combination, CPDA and citric acid was suitable for both *G*. *brevipalpis* and *G*. *austeni* feeding. While ACD did not negatively impact the productivity of *G*. *austeni*, it was not suitable for the rearing of *G*. *brevipalpis*. The addition of anticoagulants will simplify blood collection and make the blood more sterile, but they are expensive and will increase costs for large-scale operations. Moreover, as these anticoagulants did not significantly improve the blood quality and productivity of the colony, as indicated by the QF values, it would be more economical to use defibrinated blood in facilities that mass-produce *G*. *brevipalpis* and *G*. *austeni*.

Both *G*. *brevipalpis* and *G*. *austeni* are known to feed on bovine and porcine hosts in the wild [19,30]. Porcine blood has been used successfully for the *in vitro* feeding of *Glossina morsitans* Westwood and *G*. *austeni* colonies [30,31]. A combination of fresh/frozen bovine blood and reconstituted lyophilized porcine blood was used for a *G*. *austeni* colony in Tanzania [18]. Combinations of bovine and porcine blood as supplements in the synthetic diets resulted in survival rates between 70% and 90% for *Glossina palpalis palpalis* Robineau-Desvoidy [32]. The present study indicated that a combination of defibrinated bovine and porcine blood in a 50% / 50% combination or the feeding with either bovine or porcine blood on alternating days improved the overall *G*. *austeni* pupae production, and was therefore useful to accelerate colony growth. Although bovine blood seemed to be more appropriate for the maintenance of a *G*. *brevipalpis* colony, feeding a single source on alternating days did improve productivity. Porcine blood is therefore recommended to boost pupae production in the ARC-OVI colonies during times of low performance.

In 2014 it was indicated [33] that the major stimuli that elicit engorgement in hematophagous insects are associated with the host blood cellular fraction [34], although plasma components such as proteins and salts also contribute to the engorgement process in some insects [35,36]. In the past, some of these phagostimulants have been exploited to elicit a better engorgement response of blood feeding insects to improve productivity under artificial conditions. Especially, nucleotides were found to act as phagostimulants in a number of diverse hematophagous insect families, such as Pulicidae, Reduviidae, Cimicidae, Culicidae, Simuliidae, and Glossinidae [33–34,37–39] ATP was an effective phagostimulant in *Cimex lectularius* L adults and nymphs and resulted that more than 70% of these bedbugs being fully engorged [33]. The same engorgement was seen in the fleas *Xenopsylla cheopis* Rothschild and *Xenopsylla astia* Rothschild [39].

It is known that taste receptors in tsetse assist in ingestion of blood and are stimulated by ATP, ADP and AMP [37]. ATP (10^−3^ M) has been used to promote *Glossina morsitans morsitans*, *G*. *p*. *palpalis* and *Glossina tachinoides* Westwood engorgement and thus improve colony production [15,40,41]. Other nucleotides such as AMP, ADP, as well as mono- and tri-phosphates of inosine (IMP, ITP), guanosine (GMP, GTP) and cytosine (CMP, CTP) at a concentration of 10^−4^ M were effective phagostimulants for *G*. *brevipalpis* and *G*. *austeni* [23]. In the current study, increasing the concentration of ATP to 10^−3^ M significantly improved production of *G*. *austeni*. It was showed [38] that adding ATP at a concentration 10^−3^ M improved production and increased pupal weight of *G*. *morsitans*. This author also showed that increasing the concentration of ATP to 5 x 10^−3^ M did not improve reproductive rate or pupal weight in the parental generation and that ATP concentration as such is not a limiting factor for optimal feeding in an *in vitro* system.

Also, *G*. *tachinoides* Westwood was highly sensitive to ATP even at a low dose and the simultaneous use of ATP and sodium bicarbonate synergistically enhanced the feeding response [41]. These responses were, however, only achieved when a suitable feeding membrane was used [41].

The lack of significant improvement in the production of the *G*. *brevipalpis* colony in the current experiments indicate that these flies are successfully adapted to the *in vitro* feeding system currently in use, which renders the use of phagostimulants superfluous. However, wild caught adults are not adapted to an *in vitro* feeding system, and phagostimulants might increase their feeding response and productivity. Phagostimulants are expensive components and adding these routinely to the maintenance diet of tsetse flies [41], especially in mass-rearing facilities, will be very costly, and their use is only recommended when wild-collected tsetse flies experience difficulties to adapt to artificial feeding conditions.

The relatively high insemination rates and spermathecae fill in the females obtained throughout the study indicates that anticoagulants, phagostimulants or blood source did not influence the mating performance of the males.

The mass-rearing of tsetse remains challenging, especially when more than one species is involved. The optimal rearing diet may differ between colonies and tsetse species and might need to be customised for each production unit. Decisions on the most suitable rearing diet will not only depend on the biological requirements of the flies involved but will also be influenced by the availably of a suitable blood source on a continuous and economic basis. Quality control and research on factors to optimise the diet needs to be done continuously.
